# Bis[2-(hy­droxy­imino­meth­yl)phenolato]nickel(II): a second monoclinic polymorph

**DOI:** 10.1107/S1600536811001383

**Published:** 2011-01-15

**Authors:** Julia A. Rusanova, Elena A. Buvaylo, Eduard B. Rusanov

**Affiliations:** a National Taras Shevchenko University, Department of Chemistry, Volodymyrska str. 64, 01033 Kyiv, Ukraine; b Inst. of Organic Chemistry, Acad. Sci. Ukraine, Murmanska Str. 5, Kiev 02094, Ukraine

## Abstract

The title compound, [Ni(C_7_H_6_NO_2_)_2_], (I), is a second monoclinic polymorph of the compound, (II), reported by Srivastava *et al.* [*Acta Cryst.* (1967), **22**, 922] and Mereiter [Private communication (2002) CCDC refcode NISALO01]. The bond lengths and angles are similar in both structures. The mol­ecule in both structures lies on a crystallographic inversion center and both have an inter­nal hydrogen bond. The title compound crystallizes in the space group *P*2_1_/*c* (*Z* = 2), whereas compound (II) is in the space group *P*2_1_/*n* (*Z* = 2) with a similar cell volume but different cell parameters. In both polymorphs, mol­ecules are arranged in the layers but in contrast to the previously published compound (II) where the dihedral angle between the layers is 86.3°, in the title polymorph the same dihedral angle is 29.4°. The structure of (I) is stabilized by strong intra­molecular O—H⋯O hydrogen bonding between the O—H group and the phenolate O atom.

## Related literature

For the original monoclinic polymorph, see: Srivastava *et al.* (1967 ▶); Mereiter (2002 ▶). For background to direct synthesis, see: Nesterov *et al.* (2004 ▶, 2006 ▶); Kovbasyuk *et al.* (1998 ▶); Vassilyeva *et al.* (1997 ▶). For bond-length data, see: Allen *et al.* (1987 ▶).
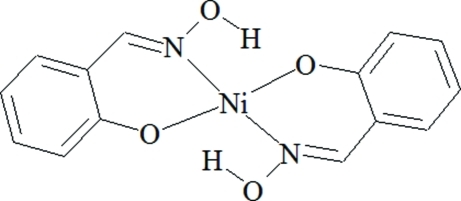


## Experimental

### 

#### Crystal data

[Ni(C_7_H_6_NO_2_)_2_]
*M* *_r_* = 330.97
Monoclinic, 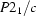

*a* = 4.9912 (2) Å
*b* = 7.4717 (3) Å
*c* = 17.4152 (7) Å
β = 90.653 (3)°
*V* = 649.42 (5) Å^3^
*Z* = 2
Mo *K*α radiation
μ = 1.51 mm^−1^
*T* = 296 K
0.53 × 0.36 × 0.11 mm

#### Data collection

Bruker SMART APEXII diffractometer
Absorption correction: numerical (*SADABS*; Sheldrick, 2002 ▶) *T*_min_ = 0.501, *T*_max_ = 0.851
4678 measured reflections
1342 independent reflections
1096 reflections with *I* > 2σ(*I*)
*R*_int_ = 0.028

#### Refinement

*R*[*F* ^2^ > 2σ(*F* ^2^)] = 0.030
*wR*(*F* ^2^) = 0.072
*S* = 1.06
1342 reflections
121 parameters
All H-atom parameters refined
Δρ_max_ = 0.28 e Å^−3^
Δρ_min_ = −0.26 e Å^−3^

### 

Data collection: *APEX2* (Bruker, 2007 ▶); cell refinement: *SAINT* (Bruker, 2007 ▶); data reduction: *SAINT*; program(s) used to solve structure: *SHELXS97* (Sheldrick, 2008 ▶); program(s) used to refine structure: *SHELXL97* (Sheldrick, 2008 ▶); molecular graphics: *SHELXTL* (Sheldrick, 2008 ▶); software used to prepare material for publication: *SHELXTL*.

## Supplementary Material

Crystal structure: contains datablocks I, global. DOI: 10.1107/S1600536811001383/br2156sup1.cif

Structure factors: contains datablocks I. DOI: 10.1107/S1600536811001383/br2156Isup2.hkl

Additional supplementary materials:  crystallographic information; 3D view; checkCIF report

## Figures and Tables

**Table:** 

N1—Ni1	1.8661 (19)
Ni1—O1	1.8292 (16)

**Table:** 

O1—Ni1—N1	93.08 (8)

**Table 2:** Hydrogen-bond geometry (Å, °)

*D*—H⋯*A*	*D*—H	H⋯*A*	*D*⋯*A*	*D*—H⋯*A*
O2—H2⋯O1^i^	0.80 (3)	1.80 (3)	2.511 (2)	146 (4)

Symmetry code: (i) 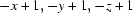.

**Fig. 1.**
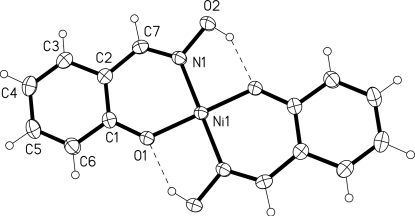
Molecular view of the title compound with the atom-labelling scheme. Displacement ellipsoids are drawn at the 30% probability level [symmetry code: 1-x;1-y;1-z].

**Fig. 2.**
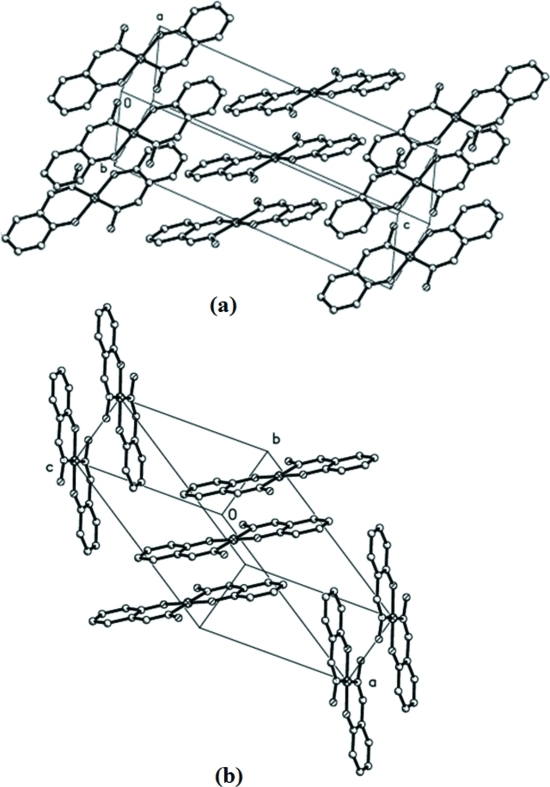
The crystal packing of the title compound (a) and compound (II) (NISALO) (b). Hydrogen atoms are omited for clarity.

## supplementary crystallographic information

### Comment

The cell dimensions of the title modification after transformation from P2_1_/c to P2_1_/n setting, are: a=18.062, b=7.472, c=4.991, β =105.39, whereas the cell dimensions of the reported compound (II) monoclinic P2_1_/n modification are: a = 13. 830, b = 4.880, c = 10.200 Å; β = 110.43° ( Srivastava *et al.*, 1967). The asymmetric unit of the title compound contains half molecule (Fig. 1), lying across a crystallographic inversion centre. The bond lengths and angles are within normal ranges (Allen *et al.*, 1987) and are comparable with the structure of the compound (II).

### Experimental

The title compound was prepared by direct synthesis: manganese powder (0.06 g, 1 mmol), Ni(OAc)_2_ (0.25 g, 1 mmol), salicylic aldehyde (0.21 ml, 2 mmol), NH_2_OH^.^HCl (0.14 g, 2 mmol), dimethylformamide (20 ml) were heated to 323–333 K and stirred magnetically for 40 min, until total dissolution of the manganese powder was observed. The transparent brown solution was allowed to stand at room temperature and brown-green crystals of the title compound suitable for X-ray analysis precipitated within few days. They were collected by filter-suction, washed with dry Pr^i^OH and finally dried in *vacuo* at room temperature (yield; 0.12 g)

### Refinement

The hydrogen atoms were located in difference Fourier synthesis and refined in isotropic aproximation.

### Crystal data

**Table:**

[Ni(C_7_H_6_NO_2_)_2_]	*F*(000) = 340
*M**_r_* = 330.97	*D*_x_ = 1.693 Mg m^−^^3^
Monoclinic, *P*2_1_/*c*	Mo *K*α radiation, λ = 0.71073 Å
Hall symbol: -P 2ybc	Cell parameters from 1800 reflections
*a* = 4.9912 (2) Å	θ = 2.3–26.1°
*b* = 7.4717 (3) Å	µ = 1.51 mm^−^^1^
*c* = 17.4152 (7) Å	*T* = 296 K
β = 90.653 (3)°	Prizm, green
*V* = 649.42 (5) Å^3^	0.53 × 0.36 × 0.11 mm
*Z* = 2	

### Data collection

**Table:**

Bruker SMART APEXII diffractometer	1342 independent reflections
Radiation source: fine-focus sealed tube	1096 reflections with *I* > 2σ(*I*)
graphite	*R*_int_ = 0.028
φ and ω scans	θ_max_ = 26.4°, θ_min_ = 2.3°
Absorption correction: numerical (*SADABS*; Sheldrick, 2002)	*h* = −6→6
*T*_min_ = 0.501, *T*_max_ = 0.851	*k* = −9→8
4678 measured reflections	*l* = −19→21

### Refinement

**Table:**

Refinement on *F*^2^	Primary atom site location: structure-invariant direct methods
Least-squares matrix: full	Secondary atom site location: difference Fourier map
*R*[*F*^2^ > 2σ(*F*^2^)] = 0.030	Hydrogen site location: inferred from neighbouring sites
*wR*(*F*^2^) = 0.072	All H-atom parameters refined
*S* = 1.06	*w* = 1/[σ^2^(*F*_o_^2^) + (0.030*P*)^2^ + 0.3216*P*] where *P* = (*F*_o_^2^ + 2*F*_c_^2^)/3
1342 reflections	(Δ/σ)_max_ = 0.005
121 parameters	Δρ_max_ = 0.28 e Å^−^^3^
0 restraints	Δρ_min_ = −0.26 e Å^−^^3^

### Special details

**Table:**

Experimental. Numerical absorption corrections based on indexed crystal faces were applied using the Crystal Faces plugin in Bruker *APEX2* software (Bruker, 2007)
Geometry. All e.s.d.'s (except the e.s.d. in the dihedral angle between two l.s. planes) are estimated using the full covariance matrix. The cell e.s.d.'s are taken into account individually in the estimation of e.s.d.'s in distances, angles and torsion angles; correlations between e.s.d.'s in cell parameters are only used when they are defined by crystal symmetry. An approximate (isotropic) treatment of cell e.s.d.'s is used for estimating e.s.d.'s involving l.s. planes.
Refinement. Refinement of *F*^2^ against ALL reflections. The weighted *R*-factor *wR* and goodness of fit *S* are based on *F*^2^, conventional *R*-factors *R* are based on *F*, with *F* set to zero for negative *F*^2^. The threshold expression of *F*^2^ > σ(*F*^2^) is used only for calculating *R*-factors(gt) *etc*. and is not relevant to the choice of reflections for refinement. *R*-factors based on *F*^2^ are statistically about twice as large as those based on *F*, and *R*- factors based on ALL data will be even larger.

### Fractional atomic coordinates and isotropic or equivalent isotropic displacement parameters (Å^2^)

**Table:**

	*x*	*y*	*z*	*U*_iso_*/*U*_eq_	
C1	0.0820 (4)	0.3351 (3)	0.41130 (12)	0.0363 (5)	
C2	0.0261 (5)	0.4911 (3)	0.36993 (13)	0.0383 (5)	
C3	−0.1766 (5)	0.4888 (4)	0.31268 (15)	0.0471 (6)	
C4	−0.3212 (6)	0.3375 (4)	0.29774 (16)	0.0540 (7)	
C5	−0.2652 (6)	0.1845 (4)	0.33888 (16)	0.0530 (7)	
C6	−0.0674 (5)	0.1815 (4)	0.39413 (15)	0.0482 (6)	
C7	0.1642 (5)	0.6566 (3)	0.38506 (14)	0.0415 (6)	
H3	−0.208 (5)	0.586 (3)	0.2867 (14)	0.040 (7)*	
H4	−0.455 (6)	0.342 (4)	0.2600 (17)	0.071 (9)*	
H5	−0.369 (6)	0.088 (4)	0.3311 (16)	0.064 (9)*	
H6	−0.029 (5)	0.081 (4)	0.4202 (15)	0.048 (7)*	
H7	0.106 (5)	0.757 (3)	0.3582 (13)	0.037 (6)*	
H2	0.578 (7)	0.828 (5)	0.4672 (18)	0.074 (11)*	
N1	0.3534 (4)	0.6733 (3)	0.43480 (11)	0.0386 (4)	
Ni1	0.5000	0.5000	0.5000	0.03283 (15)	
O1	0.2671 (3)	0.3269 (2)	0.46661 (9)	0.0413 (4)	
O2	0.4542 (4)	0.8474 (3)	0.43860 (12)	0.0586 (5)	

### Atomic displacement parameters (Å^2^)

**Table:**

	*U*^11^	*U*^22^	*U*^33^	*U*^12^	*U*^13^	*U*^23^
C1	0.0337 (11)	0.0407 (13)	0.0346 (12)	−0.0023 (10)	0.0046 (9)	−0.0038 (10)
C2	0.0372 (11)	0.0425 (12)	0.0355 (12)	0.0022 (11)	0.0022 (9)	−0.0003 (11)
C3	0.0458 (14)	0.0516 (16)	0.0436 (14)	0.0049 (14)	−0.0054 (11)	0.0030 (13)
C4	0.0467 (15)	0.069 (2)	0.0457 (15)	−0.0028 (15)	−0.0113 (12)	−0.0060 (15)
C5	0.0522 (16)	0.0531 (17)	0.0536 (16)	−0.0145 (14)	−0.0064 (13)	−0.0100 (14)
C6	0.0537 (16)	0.0421 (15)	0.0488 (15)	−0.0064 (13)	−0.0030 (12)	−0.0010 (12)
C7	0.0470 (14)	0.0384 (13)	0.0391 (13)	0.0027 (11)	−0.0033 (11)	0.0092 (11)
N1	0.0450 (11)	0.0306 (10)	0.0401 (10)	−0.0052 (9)	−0.0023 (9)	0.0034 (9)
Ni1	0.0366 (2)	0.0295 (2)	0.0324 (2)	−0.00221 (19)	−0.00104 (15)	0.00314 (18)
O1	0.0474 (10)	0.0329 (9)	0.0434 (9)	−0.0062 (7)	−0.0100 (7)	0.0056 (7)
O2	0.0717 (14)	0.0338 (10)	0.0698 (13)	−0.0151 (9)	−0.0272 (11)	0.0166 (9)

### Geometric parameters (Å, °)

**Table:**

C1—O1	1.328 (3)	C5—C6	1.371 (4)
C1—C2	1.397 (3)	C5—H5	0.90 (3)
C1—C6	1.399 (3)	C6—H6	0.90 (3)
C2—C3	1.412 (3)	C7—N1	1.280 (3)
C2—C7	1.439 (3)	C7—H7	0.93 (2)
C3—C4	1.365 (4)	N1—O2	1.396 (3)
C3—H3	0.87 (2)	N1—Ni1	1.8661 (19)
C4—C5	1.376 (4)	Ni1—O1	1.8292 (16)
C4—H4	0.93 (3)	O2—H2	0.80 (3)
			
O1—C1—C2	123.1 (2)	C5—C6—C1	121.0 (3)
O1—C1—C6	118.8 (2)	C5—C6—H6	121.2 (17)
C2—C1—C6	118.1 (2)	C1—C6—H6	117.8 (18)
C1—C2—C3	119.4 (2)	N1—C7—C2	123.8 (2)
C1—C2—C7	122.0 (2)	N1—C7—H7	119.1 (15)
C3—C2—C7	118.5 (2)	C2—C7—H7	117.1 (15)
C4—C3—C2	121.2 (3)	C7—N1—O2	112.7 (2)
C4—C3—H3	120.3 (17)	C7—N1—Ni1	128.70 (18)
C2—C3—H3	118.6 (17)	O2—N1—Ni1	118.62 (15)
C3—C4—C5	119.0 (3)	O1^i^—Ni1—O1	180.0
C3—C4—H4	118 (2)	O1^i^—Ni1—N1	86.92 (8)
C5—C4—H4	123 (2)	O1—Ni1—N1	93.08 (8)
C6—C5—C4	121.3 (3)	N1^i^—Ni1—N1	180.00 (9)
C6—C5—H5	120.1 (19)	C1—O1—Ni1	129.22 (15)
C4—C5—H5	118.5 (19)	N1—O2—H2	98 (3)

Symmetry codes: (i) −*x*+1, −*y*+1, −*z*+1.

### Hydrogen-bond geometry (Å, °)

**Table:**

*D*—H···*A*	*D*—H	H···*A*	*D*···*A*	*D*—H···*A*
O2—H2···O1^i^	0.80 (3)	1.80 (3)	2.511 (2)	146 (4)

Symmetry codes: (i) −*x*+1, −*y*+1, −*z*+1.
